# HR*func*: a tool for modeling hemodynamic response variability in fNIRS

**DOI:** 10.1117/1.NPh.13.S1.S17801

**Published:** 2025-11-20

**Authors:** Denny Schaedig, Megan Schumer, Bedilia Mata-Centeno, Luca Pollonini, Koraly Pérez-Edgar, Nadine Melhem, Susan B. Perlman

**Affiliations:** a Washington University in St. Louis, School of Medicine, St. Louis, Missouri, United States; b University of Houston, Department of Engineering Technology, Houston, Texas, United States; c The Pennsylvania State University, Department of Psychology, State College, Pennsylvania, United States; d University of Pittsburgh, School of Medicine, Pittsburgh, Pennsylvania, United States

**Keywords:** functional near-infrared spectroscopy, hemodynamic response function, deconvolution, tool, database

## Abstract

**Significance:**

Neural activation in functional near-infrared spectroscopy (fNIRS) signals is inherently convolved with, and temporally blurred by, a hemodynamic response function (HRF). Accurately modeling HRF variability during deconvolution improves neural activity recovery.

**Aim:**

We present the Python-based HRfunc tool for estimating local HRF distributions and neural activity from fNIRS through deconvolution. HRFs are stored within a tree and a hash table hybrid data structure for efficient spatial and contextual identification of relevant HRFs.

**Approach:**

To test the HRfunc tool, we conducted two analyses with hemoglobin and estimated neural activity, a general linear model (GLM) analysis on a single subject, child executive function task (n=79), and a neural synchrony analysis assessing wavelet coherence between child–parent dyads (92 dyads).

**Results:**

Estimated HRFs contained a generally canonical shape. Within estimated neural activity, kurtosis increased, skew remained stable, and signal-to-noise ratio decreased. Neural synchrony lateralization effects emerged, and consistent GLM outcomes were observed.

**Conclusions:**

These results support the use of the HRfunc tool for estimating event-based HRFs and neural activity in fNIRS studies. Through collective sharing of HRFs, an HRF database will be established to provide access to estimated HRFs across brain regions, subject ages, and experimental contexts.

## 1 Introduction

Functional near-infrared spectroscopy (fNIRS) is a noninvasive neuroimaging technique that measures changes in oxygenated (HbO) and deoxygenated hemoglobin (HbR) concentrations, providing an indirect proxy of neural activity.^1^^,^^2^ Changes in hemoglobin are physiologically linked to synaptic activity through neurovascular coupling, a relationship modeled through the hemodynamic response function (HRF), which describes the temporal coupling between neural activity and hemodynamic changes.^3^ However, the convolved hemodynamic response to neural activity is physiologically delayed, which temporally offsets and smooths hemoglobin signals relative to their proximal neural activity.^4^ In addition, the hemodynamic response varies temporally, if not altogether morphometrically, across brain regions,^5^ research subjects,^6^ and experimental contexts.^7^ Figure S1 in the Supplementary Material summarizes a history of these critical research insights.

Methods of accounting for this variability with deconvolution in functional magnetic resonance imaging (fMRI) have been adapted to fNIRS,^8^ yet tools for modeling and communicating this variability are not widely available. In the absence of a tool that meaningfully shares observed hemodynamic response variability within a sample, nuanced changes in HRF morphometry across a diverse population may be difficult to model without large-scale studies. Consequently, subtler effects within results in smaller subject pools risk being lost in the absence of an HRF model reflective of the subject pool being sampled. Therefore, a tool is needed that deconvolves HRFs and neural activity while aggregating hemodynamic response variability observed by the neuroimaging community into a scalable database to preserve observed hemodynamics for future analysis. To accomplish this, we leveraged standard deconvolution techniques alongside hybrid data structures that inherently provide quick searches across brain regions and experimental contexts. We present HRfunc, a tool for exploring HRF variability in fNIRS, and demonstrate the HRfunc tool’s feasibility by testing existing datasets.

To approximate neural signals from fNIRS data, researchers commonly use convolution to support their models, treating the stimulus time series and the HRF as input functions to generate predicted neural activation.^9^ In event-related general linear model (GLM) analyses, modeled neural events (often delta- or boxcar-shaped impulses) are convolved with a canonical HRF model to generate regressors, which are then included in the design matrix for regression analysis.^10^ Conversely, deconvolution techniques aim to invert the convolution of neural activity with the HRF, given the convolved hemoglobin concentration signals, and estimate the latent HRF and neural activity that best reproduces the observed data.^11^ Deconvolution is essential in various analysis methods, such as in machine learning, where modeling an HRF and hemoglobin is difficult, if not impossible.^12^ Furthermore, it is important to consider that convolved hemoglobin signals, such as those collected by fNIRS and fMRI, are temporally smoothed by the HRF, and fine-grained temporal patterns may be obscured. As a result, even artificial intelligence (AI) models with memory mechanisms may fail to learn important patterns from convolved hemoglobin signals that might be discernible in deconvolved neural activity.^13^ Analysis of hemoglobin using GLMs and AI, along with other analysis methods, is further complicated by the HRFs variability across brain regions,^7^ neurodevelopmental stage,^14^ and experimental context^15^; all of which can degrade the temporal alignment of the modeled or recovered neural signal if the estimated HRF does not match the true HRF.^16^

To address hemodynamic response variability, we developed an HRfunc companion open-source database called HRtree that leverages a hybrid tree–hash table data structure for efficient estimation, storage, and retrieval of HRFs. To further support our understanding of HRF variability, HRF estimates are stored as a probabilistic function within the HRtree, preserving subject-wise channel estimates and the average estimate across a subject pool, alongside subject spread. This will enable us to capture regional and contextual variability that can be leveraged to better estimate neural activity. This framework facilitates the sharing and reuse of HRF estimates across studies, enabling researchers to contribute to, and benefit from, a collective resource for modeling HRFs across brain regions and experiment contexts.

We expect that this strategy will reduce assumptions about HRF properties and, as a result, produce higher accuracy estimates of neural activity. Storing the HRF itself in a probabilistic framework will also enable long-term HRF region of interest (ROI) meta-analysis as well as data augmentation for machine learning. To validate this novel preprocessing tool for deconvolving latent HRF and neural activity estimates, we examined the tool’s impact on traditional analysis as well as signal properties within the hemoglobin and latent neural activity estimated within. We conducted two separate analyses based on block and event-based task designs. We used existing laboratory datasets examining child neural activity, observed to have variable hemodynamics from the canonical HRF,^17^^,^^18^ to test the utility of the HRfunc tool in modeling hemodynamic variability in two popular experimental contexts in developmental neuroimaging: (1) single-subject testing of cognitive function in the preschool age^19^^–^^21^ and (2) dyadic neural synchrony between parents and children.^22^^–^^24^ Data are presented to demonstrate areas of data quality increase, decrease, and/or stability through the use of the HRfunc tool.

## 2 Materials and Methods

### 2.1 Tool Methodology

Below, we outline the process for estimating and communicating HRFs and latent neural activity.

#### 2.1.1 Toeplitz deconvolution

To estimate an underlying function convolved with another, such as an HRF convolved with neural activity within HbO and HbR hemoglobin signal, Toeplitz deconvolution with Moore–Penrose pseudoinversion^25^^,^^26^ and Tikhonov regularization is utilized. The equation is defined formally below, where H is the Toeplitz design matrix, L is the regularization matrix, λ is the regularization hyperparameter, y is the fNIRS signal observed, and x represents our latent HRF or neural activity for which we are solving. Toeplitz deconvolution employs linear inversion to recover a function,^27^^,^^28^ which is advantageous compared with other methods due to its efficient computation and storage,^29^ interpretability,^30^ and integration of regularization for handling noise within a signal^29^

$$x=(H^{T}H+\lambda L^{T}L{)}^{-1}H^{T}y$$

Deconvolution is used twice within the HRfunc tool, first to estimate the underlying HRF within each subject’s fNIRS recordings, shown in Fig. S2 in the Supplementary Material. Then, underlying neural activity is estimated utilizing the HRF estimated in the first deconvolution step that reflects the subject pool and experimental paradigm. Channels that deconvolution failed to converge were dropped, assessed using a combination of condition number thresholding and computational time limits. Both the hemoglobin signal and estimated HRF are scaled automatically by the HRfunc tool, as described in Sec. 2.1.7, prior to deconvolution. The deconvolved HRF average and spread is estimated using event-wise HRF estimates across all subjects, as shown in Figs. 3 and 4. A stable regularization that minimized noise artifacts and suppression of the latent HRF was found with lambda = 1.0; the HRfunc tool defaults to this value; however, it can be tuned to modulate noise suppression.

A common artifact of Toeplitz deconvolution is highly variable edges of the estimate, as shown in Fig. S3 in the Supplementary Material, with the most accurate estimations occurring in the center of the estimate. To remove these artifacts, HRfunc implements an edge expansion process prior to deconvolution of the HRF and then trims the expanded edges post-HRF estimation. As part of this process, the events and duration used to estimate the HRF are shifted to mimic an expanded edge (defaulting to +25%) around the true HRF position prior to deconvolution. To accomplish this, the events passed into the HRfunc tool are shifted back in time by the edge expansion parameter, and the duration of the HRF is increased to account for time added to each edge of the HRF. Post deconvolution, the HRF is extracted from the expanded HRF space, largely removing the Toeplitz edge artifacts, given that the edges are sufficiently expanded.

#### 2.1.2 Communicating HRFs

Using a standardized format of storing HRF estimates within a JSON object, the HRfunc tool can easily save, load, and merge HRF estimates from different montages. As a result, researchers can load montage-specific HRF estimates or load in a large database of HRFs, such as the HRtree, and model hemodynamic responses with higher accuracy using HRFs estimated from contextually relevant experimental paradigms. Each HRF has a rich amount of context stored as an entry in the JSON object with DOI and channel as its key to prevent clashes, and all other details provided a part of its submission as a standardized dictionary. HRfunc automatically stores necessary information for communicating HRF estimates, such as HRF duration, fNIRS sampling frequency, and experiment context. This enables resampling HRFs to new montages with different sampling frequencies via spline interpolation, as described in Sec. 2.1.6. As a result of standardized formatting and information preservation during estimation, fNIRS researchers can send and receive HRF estimates they calculate to their collaborators, and the wider neuroimaging community, to leverage in their own preprocessing and analysis.

#### 2.1.3 Collaborative HRF sourcing through the HRtree database

Three steps enable the user to participate in the collaborative sharing of HRFs through the HRtree using the HRfunc tool. First, the user must estimate channel-wise HRF’s across a subject pool using the HRfunc tool. Second, the user must publish a paper or preprint and obtain a DOI to include in HRF estimate submission. This requirement is designed to detail how the data were collected and offer transparency in the origination of each HRF in the HRtree. Thus, the HRfunc tool will be comprehensive and in sync with current scientific standards. Finally, the user must submit the estimated HRFs by uploading the outputted HRFs.json, alongside their DOI and experimental contexts that reflect the data from which the HRF was estimated (e.g., task and age). HRFs estimated from subsets of subjects, reflecting a unique population within a subject pool (i.e., exclusively women, ages 10 to 20), can be submitted using the same DOI. After review of the HRF submission, the user’s estimated HRFs will be merged into the HRtree and disseminated for other users to access through the HRfunc tool. Collaborative HRF sourcing through the HRtree database is particularly useful in cases where HRfunc cannot generate an HRF for study subjects in experiments without an event-related design (e.g., resting state, neural synchrony; see Sec. 2.3.7). In these cases, the user can search the HRtree for similar experimental designs or subject age ranges or use the provided default HRF.

#### 2.1.4 Tree data structure for mapping HRFs to optodes

Two k-d tree data structures, storing either HbR or HbO HRF estimates, are used to efficiently store and search for pre-estimated HRFs in three-dimensional space. This enables long-term scaling of a large HRF database of pre-estimated HRFs due to the tree structures’ quick spatial lookup of O(log n) on average with O(n) worst-case runtime.^31^ By storing HRFs within tree nodes, HRfunc can quickly search x, y, and z dimensions for HRF estimates within a distance threshold containing potentially usable pre-estimated HRFs. Both a k-d tree nearest neighbor and radius search algorithm are implemented to leverage the tree data structure to find the closest HRF estimate(s) to a given optode within a given distance. If no HRF estimate can be found, a canonical double-gamma HRF is instead passed back and used for deconvolving neural activity. This enables quick searches for HRFs within a maximum distance, collection of HRFs within an ROI, and the ability to rely on standard HRF models if an HRfunc estimate is unavailable.

To enable global HRFs to be added into the HRtree, locations are assigned randomly centered around (360, 360, 360) with a max deviation of 1. This prevents their location from being mixed with location specific estimates. By introducing randomness centered outside of typical NIRS coordinates, this ensures the HRtree generates a subtree, instead of a long tail of global HRFs, and avoids worst-case runtime behavior. A canonical double-gamma HRF is always attached to the root’s right node at (359, 359, 359) and used as a default HRF if the nearest neighbor algorithm fails to locate an HRF estimate node. Attaching a canonical HRF to the right of the root ensures a global HRF cluster forms at the base of the HRtree, instead of the edges, and avoids worst-case runtime behavior.

#### 2.1.5 Hash table for storing and hashing on context

To enable experimental context to be stored and quickly searched for, each context item attached to an HRF tree node (i.e., task, age range, stimuli intensity, and DOI) is added into a hash table alongside pointers to the HRFs node in the HRtree. This structure enables quick insertions and searches in O(1) average or O(n) worst-case runtime through hashing^32^ on context and building sub-trees with similar contexts, as described in Sec. 2.2.3. The HRF hash table uses secure hashing algorithm 3 (SHA-3) alongside open-addressing with a quadratic probe to resolve collisions and minimize primary and secondary clustering.

To prevent unintentional collisions with contexts of similar forms, context is combined with its context group with a hyphen while hashing (e.g., “task-flanker” or “study-care”). Experimental contexts that include a numerical range, such as ages, are binned then hashed on each bin starting from the center of the range and moving out (e.g., age 5, age 6, age 4, and age 7). Contexts based on numbers and floats are hashed similarly to age range with bins over a range, defaulting to searching over a single bin if only one number is provided. Binning numerical experimental contexts enables the tool to iterate over a range of acceptable contexts (i.e., duration between 12.0 and 30.0 s or intensity between 0.8 and 1.0). Although hashing logic remains the same as searching through an age range from center to edges, the bin size is set to 1.0 and 0.1 for duration and stimuli intensity, respectively.

#### 2.1.6 Resizing via spline interpolation

A piecewise polynomial function, or “spline”, is used to interpolate intermediary datapoints of an HRF to resize the function temporally to the sampling rate of the fNIRS scan to be deconvolved. A spline function is calculated by solving a cubic polynomial Si(x), and once constructed, it is used to estimate the value of any datapoint along a range.^33^ This is useful as it enables calculation of intermediary datapoints along a continuous interval and ultimately interpolation of a resized HRF through a single pass.

#### 2.1.7 Scaling

Values that are extremely small or approach infinity can interfere with deconvolution.^34^ To prevent this issue while deconvolving an HRF estimate, convolved hemoglobin signal is normalized through *z*-scoring to preserve the HRF shape and provide numerical stability. Prior to deconvolving neural activity, the estimated HRF is scaled so ${\mathrm{HRF}}_{\max}=1$, preserving the respective strength of the peak and undershoot(s). In addition, a small ε value of $1\times 10^{-7}$ is added to the scaling equation to prevent extremely small values approaching 0. Scaling or attenuating extreme values prior to deconvolution provides stability in the inversion process by reducing the amplification of noise.^16^

### 2.2 Tool Functionality

**Fig. 1:**
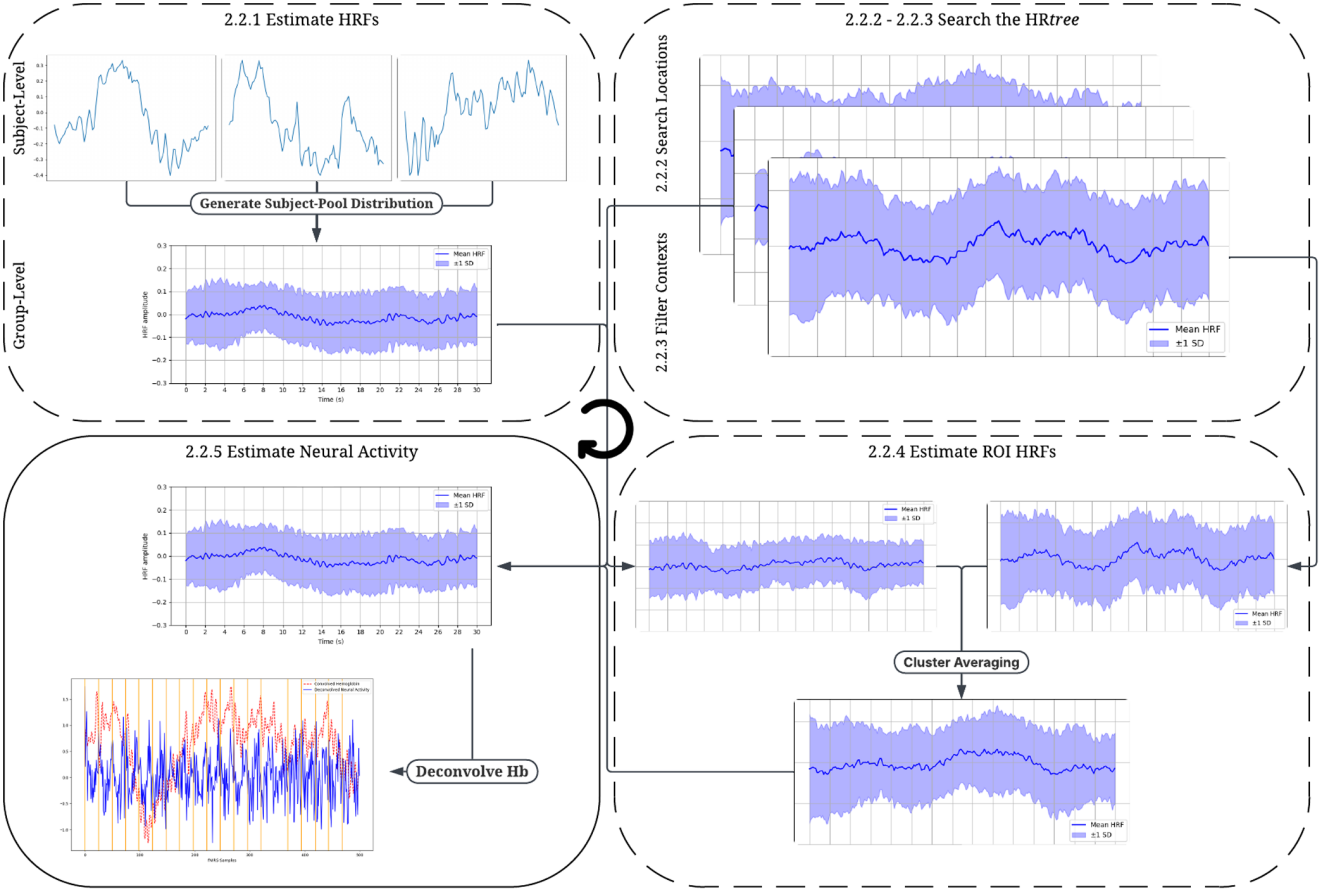
Diagram describing the workflow of HRfunc and how it relates to the following sections. Dotted boxes are optional steps a user can take when estimating neural activity. Without obtaining an estimated HRF described in Secs. 2.2.1–2.2.3, a canonical HRF is used for neural activity estimation.

The following sections describes the user-oriented functions for modeling HRFs in fNIRS. Figure 1 illustrates how the user-oriented functionality of HRfunc can be leveraged to estimate HRFs and neural activity in fNIRS. Visit www.hrfunc.org for tutorials and video guides on applying each function to fNIRS data.

#### 2.2.1 Estimating HRFs within channels

Toeplitz deconvolution, as described in Sec. 2.1.1, is used to estimate both the latent HRF and its variability across channels, subjects, and events. To estimate these, a user passes into the HRfunc tool, an MNE fNIRS object, and an event impulse series, the same length of the scan for each subject. For each fNIRS scan passed in, channel-wise HRF estimates are calculated to preserve spatial and subject differences. After deconvolving each subject HRF estimate, a subject-pool-wide HRF trimmed average and standard deviation is calculated for each channel. Subject-level HRF estimates are preserved and saved for future ROI meta-analysis, as described in Sec. 2.2.4.

#### 2.2.2 Localizing previously estimated HRFs

Using the functionality of the HRtree and HRfunc data structures, HRF estimates that spatially neighboring optodes are localized using the nearest neighbor search outlined in Sec. 2.1.4. The search function finds an HRF within a maximum distance provided (defaulting to 1 mm) to an optode using the recursive nearest neighbor algorithm, custom to k-d trees, calculating the Euclidean distance between optode and HRFs and pruning near and far branches as it progresses to reduce search space. Using this nearest neighbor search algorithm, the sub-search for the closest HRF is found in O(log n) time compared with brute nearest neighbor search of O(n). Pairing this search functionality with the experiment context filtering capacity of HRfunc, described in Sec. 2.2.3, contextually relevant local HRFs may be found. If no local HRF is found within the maximum allowed distance, a canonical double-gamma HRF estimate is used as a backup to mirror standard deconvolution approaches that use a canonical HRF.

#### 2.2.3 Finding HRFs with relevant context

Experimental contexts of interest passed into the HRfunc tool (i.e., age range, demographics, and DOI) are used to filter the HRtree, where only HRF nodes that pass a similarity threshold to the context of interest, defaulting to 95%, are inserted. In addition, leveraging the in-built functionality of hash tables, relevant context can be filtered for through the branch() function. This hashes on requested contexts building and returning a sub-tree that contains at least partially relevant HRFs with contexts requested. These filtering methods create a smaller context-specific tree that one can search through for locations or other contexts. Similarity is assessed by the weighted percent similarity of the requested context to the HRF in question. All contexts are weighted the same unless specified otherwise, enabling efficient and highly specific context searches, as well as contextless searches that only focus on the location of the optode.

#### 2.2.4 Estimating HRFs across regions of interest

A radius search algorithm is implemented, as described in Sec. 2.1.4, which enables a radius search around a particular optode location that returns all HRF estimates within the ROI. Alongside each channel-wise HRF estimate, the intermediary subject-level HRF estimates are automatically preserved and saved by the HRfunc tool. This enables calculation of an average HRF across an ROI and provides a framework for ROI analysis on multiple subject-pools from similar, yet different, experimental contexts and montages. Although only a radius ROI search is integrated into the HRfunc tool, more complex ROI search algorithms that leverage the k-d tree and hash table data structure could easily be implemented for creating ROIs with higher contextual and spatial specificity.

#### 2.2.5 Estimating latent neural activity

**Fig. 2:**
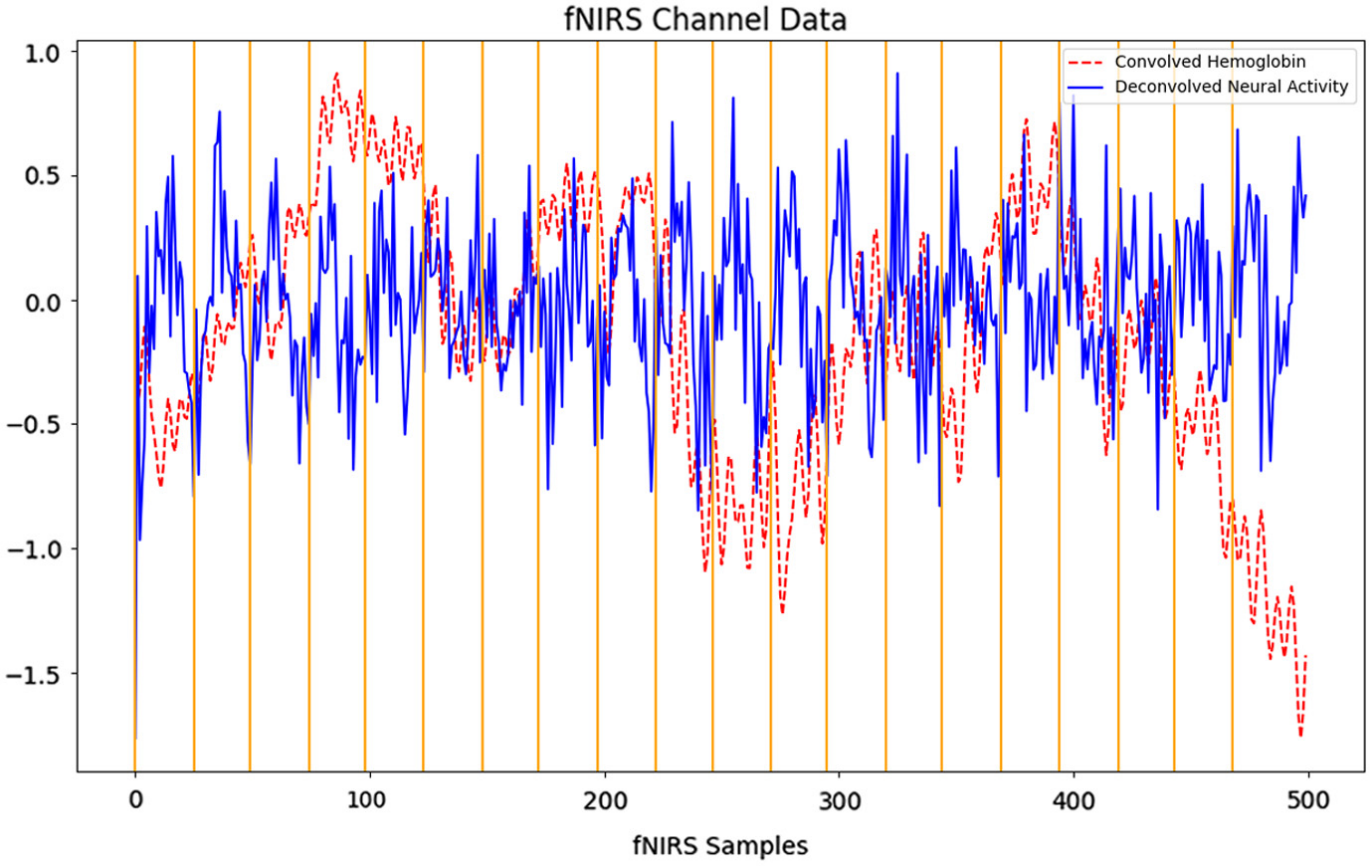
Convolved hemoglobin (mol/L units) recorded during a Flanker task and overlaid deconvolved neural activity estimates (a.u. units). Trial timepoints, latent HRFs are estimated from, are marked with vertical lines.

Using an estimated local HRF, or shared HRF estimate, channel signals are deconvolved for each subject again using deconvolution, as described in Sec. 2.1.1. This deconvolution estimates the latent neural activity in the fNIRS hemoglobin signal convolved with the HRF, as shown in Fig. 2. Prior to deconvolution, HRFs are normalized to have a maximum value of 1 while scaling negative values, such as an undershoot, across time to minimize extremely small values and prevent instability during deconvolution.^16^ The HRfunc tool applies deconvolution in place, altering an MNE NIRX object^35^ directly replacing hemoglobin (mol/L) with neural activity estimates (arbitrary units, a.u.).

### 2.3 Evaluation and Testing

Below, we briefly describe how the capabilities of the HRfunc tool were tested and the existing datasets utilized.

#### 2.3.1 P-CAT dataset

The parent–child anxiety transmission study (PCAT) is an ongoing longitudinal study designed to investigate transmission of anxiety from parent to child. We used data from the R56 dataset, in which 4- to 7-year-old children participated in multiple tasks. All participants gave informed assent alongside their parent or guardian’s consent in accordance with institutional review board (IRB) guidance and approval. Data here were taken from the custom-designed “Gooble” flanker task in which children are told the story of an alien who gets lost on Earth and must find his way home. Throughout the task, the lost alien encounters scenes (blocks) in which he is *congruent* with his scenery, such as dogs or cars (i.e., facing the same direction) or *incongruent* with his scenery (i.e., facing the opposite direction). Children must push a button to indicate the direction that the lost alien is facing. Incongruent blocks are more challenging than congruent blocks as the child must focus on the alien and ignore the scenery pointing in a different direction. Both blocks are compared with a nondirectional baseline in which scenery remains centered and does not point in any direction (e.g., trees). Usable data were collected from n=79 children. This study was approved by the Institutional Review Board at Washington University in St. Louis.

#### 2.3.2 CARE dataset

The CARE study is a longitudinal study designed to assess the role of parent support in predicting psychopathology outcomes in children experiencing parental conflict. We used data from the first study visit, in which children were 4 to 7 years old. Informed consent was obtained through assent of the children, alongside their parents or guardians’ consent, in accordance with IRB guidance and approval. Children participated with their parent in our widely used DB-DOS BioSync task, which induces mild stress during parent–child interaction.^23^ We measure interpersonal neural synchronization in n=92 parent–child dyads and assess the difference in synchrony between low-conflict and high-conflict families. This study was approved by the Institutional Review Board at Washington University in St. Louis.

#### 2.3.3 Preprocessing

To ensure consistency, fNIRS signals were preprocessed using two parallel pipelines: one optimized for traditional hemodynamic modeling, such as for GLMs with HRF regressors, and another for deconvoluting both hemodynamic responses and neural activity estimates. For both preprocessing methods, fNIRS signals were first converted from raw intensity measurements into optical density (OD) utilizing the logarithmic formulation of the modified Beer–Lambert law to account for variations in photon pathlength and tissue absorption essential for detecting hemoglobin.^36^ The scalp coupling index,^37^ peak power, and power spectral densities^38^ were then calculated using the OD, and channel signals that did not meet quality control thresholds described in Sec. 2.3.4 were excluded. To remove motion artifacts from the signal, temporal derivatives distribution repair was then conducted to detect fluctuations outside of the expected hemodynamic range and regionally smooth out artifacts.^39^

As an alternative to bandpass filtering for deconvolution preprocessing, we next removed physiological noise in the optical density signal by detrending using a first-order polynomial function. This preserves important spectrum frequencies the HRF may bleed into, below 0.01 and above 0.2 Hz.^40^ We further apply Beer–Lambert law to relate the concentration of OD to the concentration of oxygenated and deoxygenated hemoglobin.^41^ All signals are then baseline corrected as the deconvolution process assumes the signal centers around 0 and the baseline does not drift.^42^ The deconvolution pipeline proceeded with estimating neural activity by deconvolving an HRF estimate out of the convolved hemoglobin signal, as described in Sec. 2.2.5. For the standard preprocessing pipeline, hemoglobin finished preprocessing with a bandpass filter between the frequencies 0.01 and 0.2 Hz to remove physiological artifacts. Notably, bandpass filtering the signal prior to deconvolution is not recommended due to its dephasing effect and removal of important hemodynamic frequencies latent the HRF.^36^

#### 2.3.4 Data quality control

The signal-to-noise ratio (SNR) was calculated through a power spectrum density (PSD), with signal defined as frequencies between 0.01 and 0.2 Hz, the frequency range in which the HRF lies,^43^ and noise defined as frequencies outside this range (>0.2 and <0.01  Hz). Scans with an SNR<5.0 were excluded from analysis. The scalp coupling index (SCI) was used to assess the quality of the optode connection to participants’ scalps as the fNIRS recording was made.^37^ Channels with an SCI of <0.95 were excluded. To further compliment the SCI, we visually verified that the cardiac frequency band was present within the expected frequency range of 0.8 to 1.5 Hz^44^ to ensure biological signals were captured and excluded those without. Peak power for each scan was assessed as another measurement of signal to noise, where a low value indicates noise overpowering physiological rhythms and high values indicating nonphysiological artifacts such as motion artifacts.^38^ Peak spectral density (PSD) was visually checked for the presence of cardiac, respiratory, and task-evoked hemodynamics within their frequency range,^45^ and subjects significantly absent of these signals were excluded.

#### 2.3.5 Signal kurtosis and skewness

To assess the impact of deconvolving the convolved hemoglobin signal to estimate neural activity, the skew and tailedness of the signal were computed using the third and fourth central moments of the resulting neural activity and the convolved hemoglobin signal it originated from. A skewness value of 0 indicates a symmetric distribution, whereas positive skew suggests a right-tailed distribution and negative skew indicates left-tailed distributions. A kurtosis value of 0 corresponds to a Gaussian distribution, where values higher than 0 suggest a sharper peak and heavier tails, known as a leptokurtic distribution. Kurtosis values less than 0 indicate flatter peak and lighter tails known as a platykurtic distribution.^46^ To assess the statistical significance of changes in signal kurtosis and skew, a two-tailed paired t-test was conducted on global kurtosis, skew and signal-to-noise between convolved hemoglobin and deconvolved neural activity.

#### 2.3.6 GLM examining congruent versus incongruent

To assess the impact of deconvolution of hemoglobin signal on fNIRS analysis, a GLM analysis was performed using both traditionally preprocessed and deconvolved neural activity estimates, as described in Sec. 2.3.3, from the P-CAT studies Flanker task, described in Sec. 2.3.1. HRFs were first estimated directly from task trials using the methods described in Sec. 2.2.1, then used to estimate neural activity through deconvolution of the hemoglobin signal, as described in Sec. 2.2.5. A within-subject contrast (congruent versus incongruent) was computed using fNIRS signals acquired during the Flanker tasks’ directional trials. For analysis of convolved hemoglobin signals, the design matrix included a canonical double-gamma HRF as a regressor to compensate for the lack of hemodynamic and temporal modeling of neural activity. GLM outcomes were assessed using a repeated-measures analysis of variance (ANOVA) to assess variance between congruent and incongruent trials alongside convolved hemoglobin and deconvolved neural activity.

#### 2.3.7 Wavelet coherence synchrony

To assess the impact of estimating HRFs and neural activity on co-variance, wavelet coherence synchrony was assessed between traditionally preprocessed fNIRS hemoglobin signals and deconvolved neural activity, as described in Sec. 2.3.3, using the DB-DOS BioSync task discussed in Sec. 2.3.2. The DB-DOS BioSync does not have an event-related design, making HRF estimation impossible. Instead, the HRF estimates from the P-CAT Flanker task, described in Sec. 2.3.6, were utilized to deconvolve the CARE children’s neural activity. This is possible given the CARE child subjects’ similarity in age and demographics with the HRF estimated through the P-CAT Flanker available in the HRtree. In the absence of an HRF estimate that fit the parent’s demographics, the HRfunc tool relied on its backup canonical HRF for deconvolving the parents’ scan (i.e., the default HRF).

For each child–parent dyad, we computed the continuous wavelet transform (CWT) using a Morlet wavelet. We then combined these transformations into a third CWT and calculated wavelet coherence through a normalized squared cross-spectrum as a final metric of dyadic synchrony.^47^ Dyads without shared channels, due to channels being dropped from noise or deconvolution failing to converge, were excluded from the analysis. Neural activity synchrony was assessed on the neurogenic frequency band (0.02 to 0.05 Hz) and hemoglobin synchrony was assessed on the neurogenic + myogenic bands (0.02 to 0.15 Hz) to capture anticipated signal frequencies in each modality.^38^ Frequencies that did not contain a reliable number of cycles within task blocks were excluded from analysis (0.02 to 0.03 Hz). A one-way repeated-measures ANOVA with *post hoc* pairwise comparisons was conducted with Holm corrections for multiple comparisons to assess the difference in synchrony between blocks within the DB-DOS task. A separate two-way repeated-measures ANOVA was conducted with factors hemisphere, left versus right, and regions, dorsolateral prefrontal cortex (DLPFC) versus ventrolateral prefrontal cortex (VLPFC), on synchrony to assess spatial effects.

## 3 Results

### 3.1 HRF Estimates

**Fig. 3:**
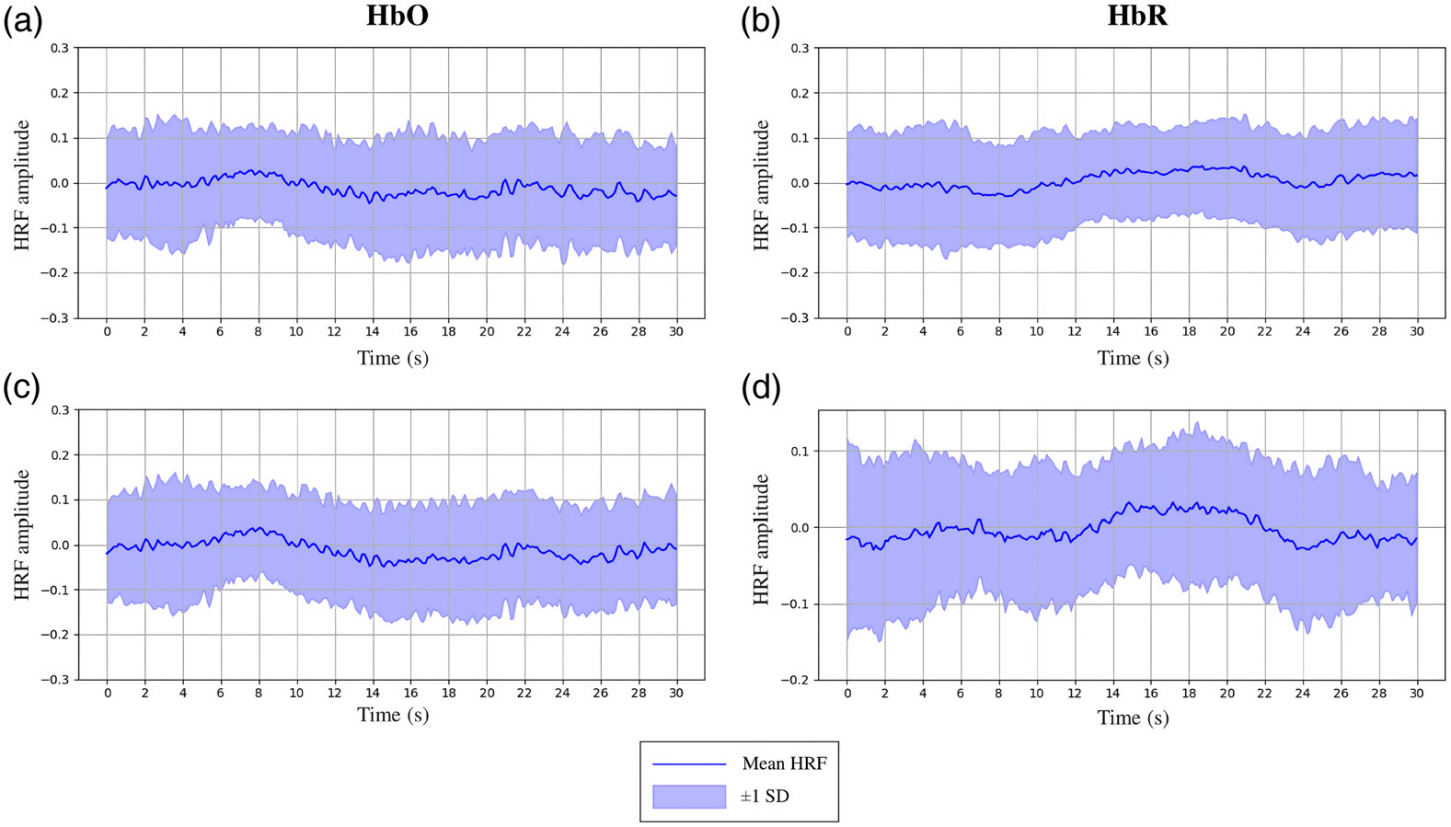
30 s HRF channel estimates from the P-CAT subject pool of 4- to 7-year-old children completing a Flanker task while recording cortical activity using fNIRS. HRFs (a) and (c) were estimated from HbO channels, and HRFs (b) and (d) from HbR channels.

**Fig. 4:**
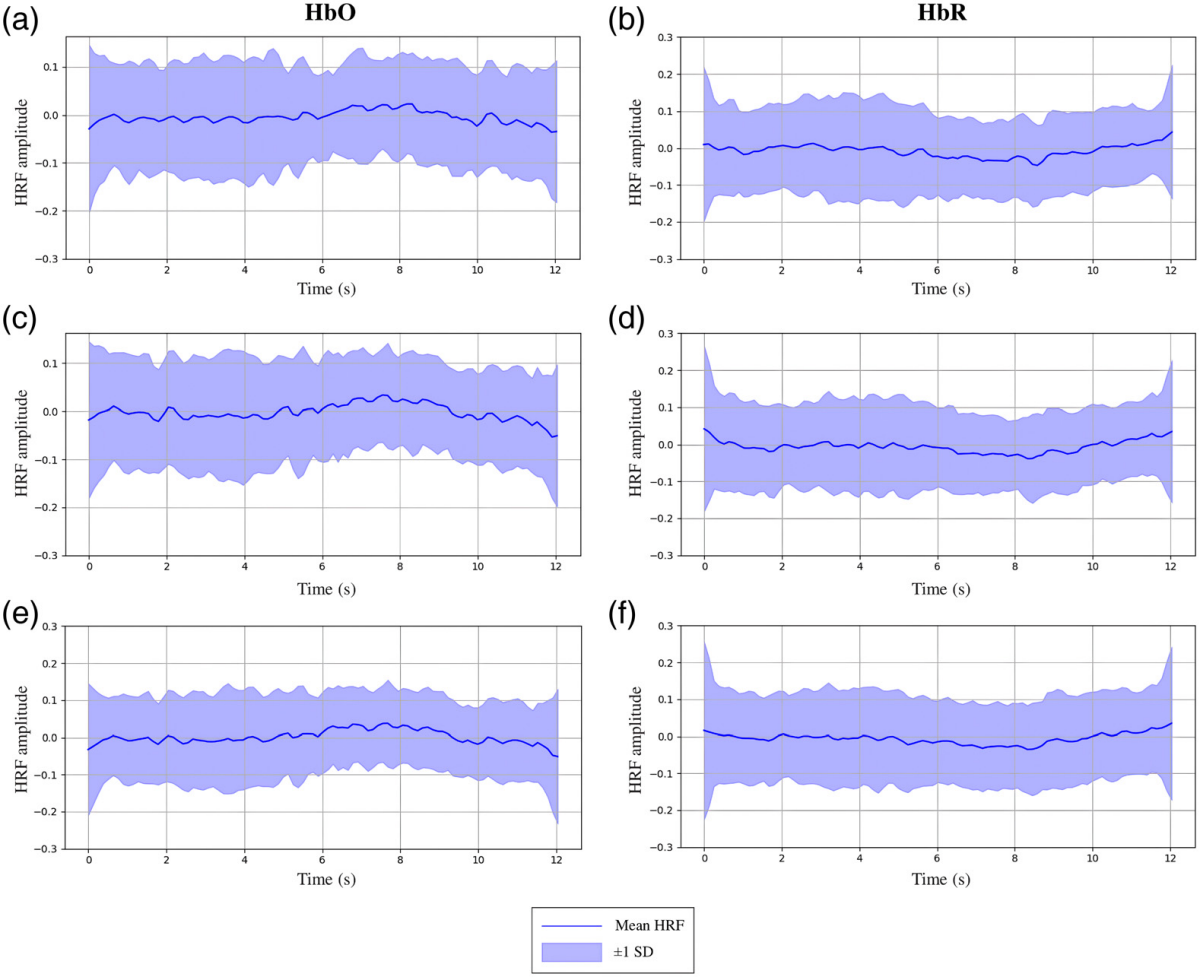
12 s HRF estimate distribution from different channels of fNIRS recordings from the P-CAT subject pool of 4- to 7-year-old children completing a Flanker task. HRFs (a), (c), and (e) were estimated from HbO channels, and HRFs (b), (d), and (f) from HbR channels.

Oxygenated channels produced an HRF, shown in Figs. 3 and 4, with a generally canonical shape; an initial undershoot was not significantly present; however, an initial peak centered around 8 s emerged across subjects, followed by a longer undershoot lasting 30 s, typical of a canonical HRF.^48^ Deoxygenated channels were generally the inverse of their colocalized oxygenated channels and appeared to have a characteristic lag. Variability between subjects was observed, as shown by the large standard deviation between subject HRF estimates in Figs. 3 and 4.

### 3.2 Deconvolutions Impact on Signal and Noise

**Fig. 5:**
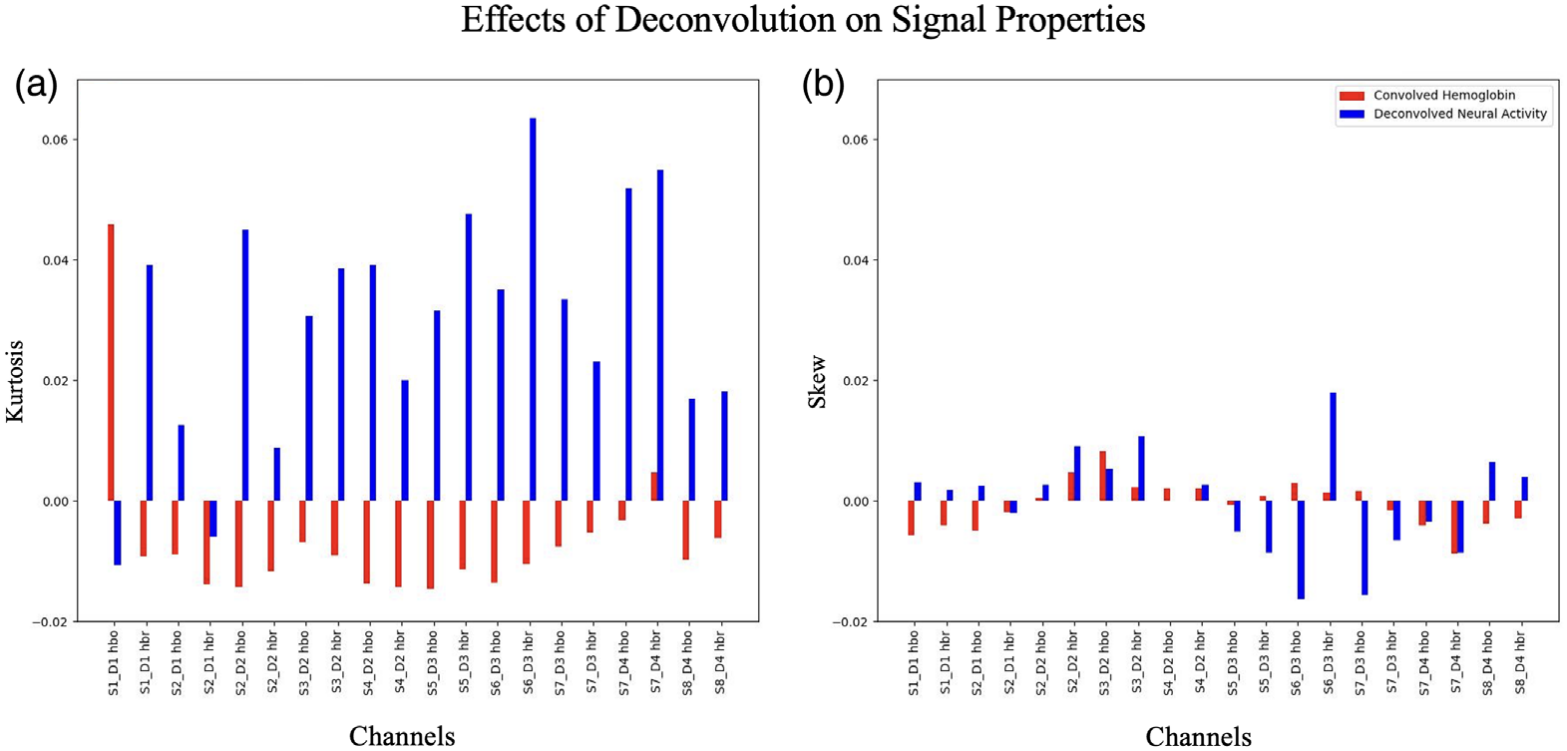
Average changes in channel signal properties, kurtosis (a) and skew (b), by deconvolution of convolved hemoglobin with estimated HRFs to deconvolved neural activity estimates across the P-CAT subject pool.

SNR in deconvolved neural activity estimates was on average 14.03 across channels within the P-CAT subject pool. This was smaller than what was observed with convolved hemoglobin signals with an SNR of 17.87, and a two-tailed paired t-test found a statistically lower SNR in deconvolved neural activity (p<0.001) compared with convolved hemoglobin. Deconvolution of hemoglobin signals had a minimal impact on the skewness of fNIRS signals; a two-tailed paired t-test revealed no significant difference between neural activity and hemoglobin skew. Deconvolved neural activity estimates appeared to show a consistent increase in kurtosis of 0.02 across channels compared with convolved hemoglobin, also shown in Fig. 5. A two-tailed paired t-test revealed a significant increase in kurtosis (p<0.001) in deconvolved neural activity compared with hemoglobin.

### 3.3 P-CAT General Linear Model

**Fig. 6:**
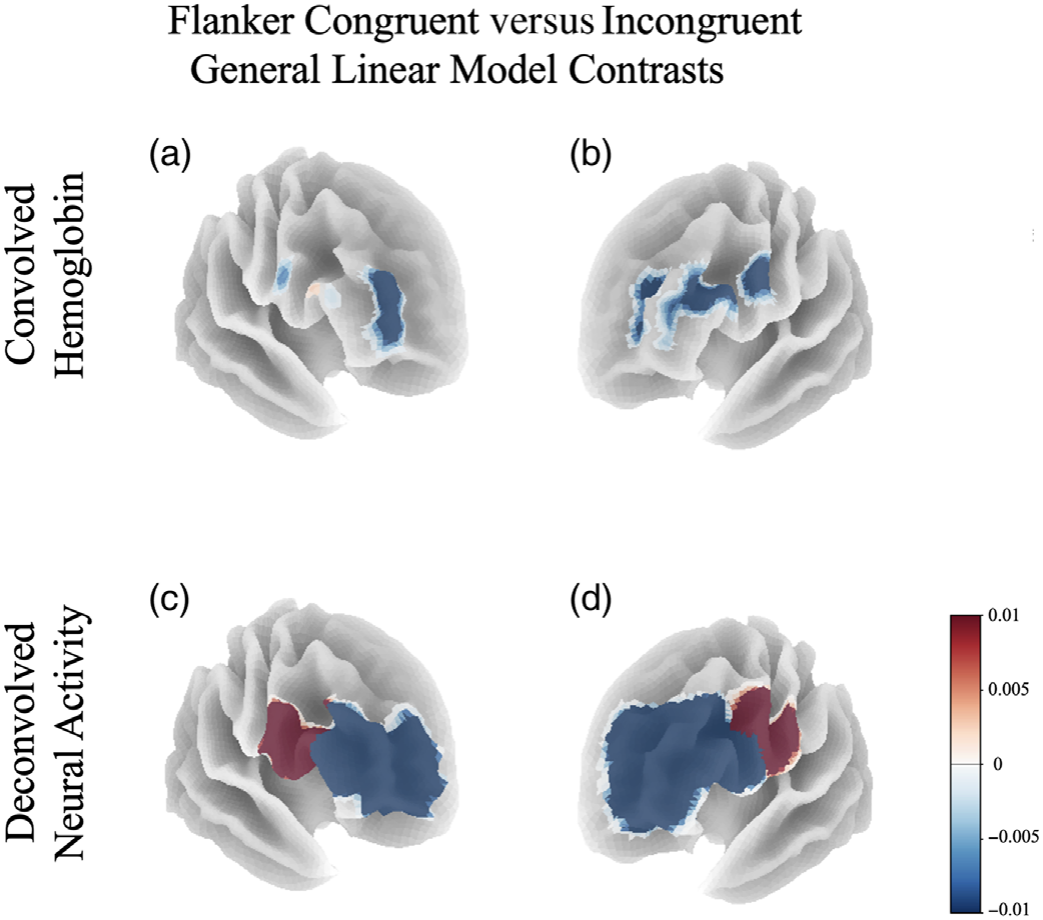
GLM congruent–incongruent contrasts results from the P-CAT Flanker fNIRS recordings, transformed into MNI space using coregistration with magnetic image resonance anatomical images and outputted into a surface statistical map within the left (a and c) and right (b and d) hemispheres for convolved hemoglobin (a and b) and neural activity (c and d) signals.

Contrast effects resulting from a GLM analysis on the deconvolved P-CAT Flanker task, described in Sec. 2.3.6, were on average 2 orders of magnitude greater than a GLM of hemoglobin with a double-gamma HRF regressor. An increase in lateralization effect of executive function was observed, as shown in Fig. 6. A repeated-measures ANOVA found significant difference in neural activity and hemoglobin between congruent and incongruent trials in channels within the right, F(1,79)=30.11, p<0.001, and left DLPFC, F(1,79)=16.82, p<0.001, alongside right, F(1,79)=10.92, p=0.003, and left VLPFC, F(1,79)=4.31, p=0.047. No significant interaction main effects between signal type × condition were observed, all F(1,79)<2.6, all p>.12. Neural activity effects trended larger, but the observed effects did not reach significance after multiple comparisons corrections.

### 3.4 CARE Synchrony

**Fig. 7:**
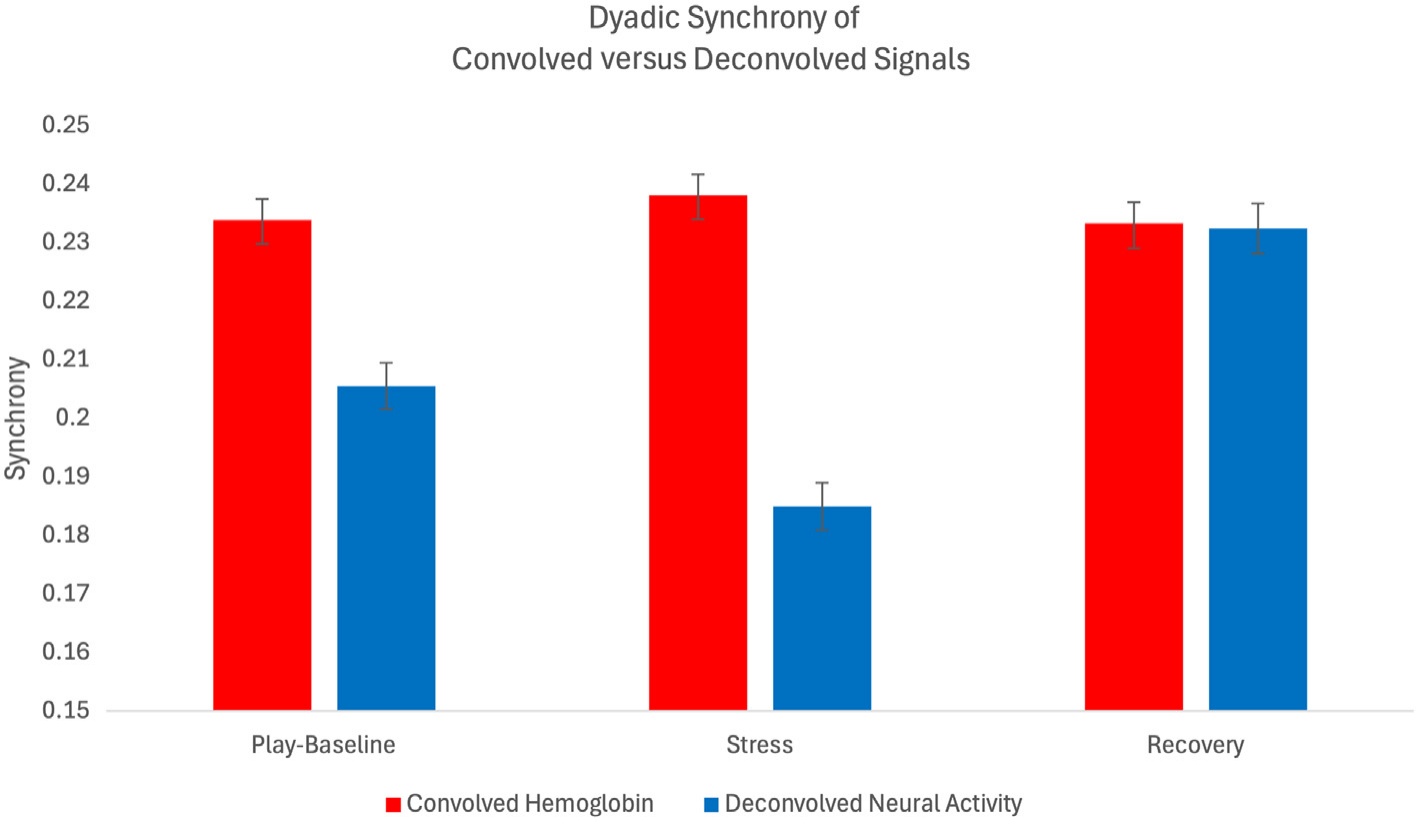
Comparison of wavelet coherence synchrony with error in the CARE study DB-DOS hyper-scanning task examining synchrony between parent–child dyads convolved hemoglobin and deconvolved neural activity signals.

Wavelet coherence synchrony of neural activity across child–parent dyads, deconvolved using the P-CAT Flanker estimated and canonical HRFs for children and adults, respectively, was 0.208 during the DB-DOS task, a 0.01 decrease in average synchrony compared with synchrony in dyads convolved hemoglobin signals (Fig. 7). A one-way repeated-measures ANOVA of synchrony within neural activity revealed a significant main effect of block, F(2,184)=57.97, p<0.001. *Post hoc* paired t-tests with Holm correction indicated that synchrony differed significantly between play-baseline versus stress blocks (p<0.001), play-baseline versus recovery blocks (p<0.001) and stress versus recovery blocks (p<0.001). When synchrony was assessed on convolved hemoglobin, a significant block effect was observed, F(2,184)=4.45, p=0.01. *Post hoc* corrections indicated synchrony differed significantly within the stress and recovery blocks (p=0.01).

A two-way repeated-measures ANOVA on synchrony within deconvolved neural activity revealed a significant effect of brain region, F(1,92)=42.31, p<0.001, and hemisphere, F(1,92)=14.19, p<0.001. The hemisphere x region interaction was also significant, F(1,92)=9.55, p=0.002, indicating the hemisphere effect was dependent on region. *Post hoc* paired t-tests with Holm correction revealed that the hemisphere difference was significant in the VLPFC (left versus right, p<0.001) but not in the DLPFC (p=0.628). In addition, higher synchrony was found in the right VLPFC versus right DLPFC (p<0.001) but not in the left VLPFC versus left DLPFC (p=0.095). A two-way repeated-measures ANOVA on hemoglobin synchrony revealed a significant effect of brain region, F(1,92)=23.27, p<0.001. *Post hoc* paired t-tests with Holm corrections revealed significant regional differences in the left DLPFC versus VLPFC (p<0.001) and the right VLPFC versus DLPFC (p=0.002). No significant difference in hemoglobin synchrony between hemispheres was observed.

## 4 Discussion

We introduce HRfunc, a novel open-source tool for estimating local and context-specific HRFs, communicating those estimates, and recovering neural activity estimates from convolved hemoglobin signals. This was accomplished through deconvolving channel-specific HRF estimates from convolved hemoglobin signals for each subject in a subject pool, then estimating a subject-pool-wide HRF for each channel. This HRF can then be used again in deconvolution to estimate the latent neural signal for each channel, removing temporal blur and gaining a closer neural signal estimate. Furthermore, this estimated HRF can continue to be used to estimate neural activity in different subject pools with similar experimental contexts. The default deconvolution method employed was Toeplitz deconvolution with Tikhonov regularization, as described in Sec. 2.1.1; however, the deconvolution function can be replaced with a custom deconvolution method that complies with MNE fNIRS objects transformation requirements.

A hybrid tree and hash table data structures are leveraged to enable long-term scaling of HRF storage, access, and communication through efficient lookup of HRFs. The tree component of the structure provides quick lookups of specific locations and incorporates nearest neighbor and radius searches. This enables finding HRF estimates from different montages with slightly different coordinates or within an ROI and establishing a regional HRF. A hash table provides quick lookups of experiment contexts, such as DOI number, task, and stimuli intensity. A custom pre-hashing logic enables hashing on ranges, with searches that radiate from the average, to find contexts within a range such as stimuli intensity (i.e., 0.8 to 1.2) and duration (12.0 to 30.0 s). The tool provides a flexible and scalable foundation for storing HRFs estimated from different montages, communicating these estimates, preprocessing of fNIRS within the MNE framework,^49^ and gaining a deeper understanding of HRF variability across brain regions and contexts.

To validate the effect of HRF and neural activity estimation with the HRfunc tool, we first compared the HRF estimates temporal profile, shown in Figs. 3 and 4, with a canonical HRF. Both shared a similar profile with an initial short peak followed by a long undershoot. The children’s estimated HRF appeared to have a small initial flatline 5 s long, uncommonly modeled with canonical HRFs. A delayed and blunted peak was observed around 8 s, which has been previously reported in children between the ages of 4 and 7,^50^ from whom the HRF was estimated. A characteristic lag compared with their collocal oxygenated channels activity appeared to form in deoxygenated channels, a result previously reported.^51^

We then assessed the changes in signal properties between deconvolved neural activity estimates and convolved hemoglobin signals. An increase in SNR to an average of 14.03 across channels and subjects was observed in deconvolved neural activity compared with the convolved hemoglobin signal it originated from, which was significantly lower compared with the standard preprocessed hemoglobin data with an SNR 17.87. This decrease may be explained by deconvolution amplification of noise within higher and lower frequency bands, typically used as noise-bands, not captured by the HRF. This suggests bandpass filtering after estimating neural activity may be necessary to reduce noise amplified during the deconvolution process. This decrease in SNR may further be explained by consistent overlap of events within the Flanker task, resulting in less accurate HRF and neural activity estimates. When HRFs overlap due to short experiment trials, this can result in high collinearity, ill-conditioned inversion, and unstable deconvolution that amplifies noise.^52^^,^^53^ Consequently, the difference in SNR between neural activity and hemoglobin concentration may provide a useful indirect metric of deconvolution accuracy. A statistically significant increase in kurtosis post deconvolution, with signal skewness remaining somewhat stable, was observed. The increase in kurtosis may suggest that deconvolution is successfully removing the temporal smoothing of the HRF and restoring the fast-spiking nature of neural activity. This appears to be further supported by the hemoglobin and neural activity characteristics shown in Fig. 2.

A GLM and wavelet coherence analysis using the P-CAT Flanker and the CARE DB-DOS dyadic synchrony task were conducted to observe the effect of deconvolution on standard fNIRS analysis. A repeated-measures ANOVA assessed on GLM outcomes revealed significant differences between congruent and incongruent trials in both hemoglobin and neural activity. These differences are consistent with the increased cognitive demand of incongruent trials due to the need for inhibitory control over conflicting stimuli.^54^ Neural activity effects compared with hemoglobin with an HRF regressor, although trending higher, did not survive corrections. The magnitude of this effect may depend on how hemodynamics are modeled, task characteristics, subject population, and statistical power.

Estimating neural activity through deconvolution of hemoglobin with a previously estimated HRF from an alternative subject pool appeared to improve analysis outcomes. Compared with standard preprocessed hemoglobin signals, synchrony between subjects deconvolved neural activity decreased on average by 0.01 across all dyadic pairs. A significant effect of interhemispheric lateralization in synchrony emerged in deconvolved neural activity. Across both hemispheres, synchrony was significantly higher in the DLPFC compared with the VLPFC. These patterns are characteristic of executive function and top-down regulation in the frontal cortex.^55^ Similar regional effects between the DLPFC and VLPFC were observed in hemoglobin synchrony; however, the lateralization effects were absent. These analysis results echo a larger body of neuroscientific findings surrounding executive function and the prefrontal cortex. This suggests that deconvolved neural activity compared with convolved hemoglobin signals may better model the brain’s activity.

The emergence of significant effects with deconvolved neural activity may be explained by individual differences in hemodynamics. The impact of age has been investigated thoroughly, and younger children typically are shown to have a significantly delayed and blunted peak.^56^^,^^57^ Theoretically, a portion of a child’s HRF peak coincides temporally with the undershoot of an adult’s HRF. As canonical HRFs attempt to model an adult HRF, this mismatch in morphometry could explain the significant difference in results between traditional GLM analysis methods modeling hemoglobin and HRF’s compared against deconvolved neural activity. Overall, our results support that estimating latent HRFs and neural activity using the HRfunc tool improves signal interpretability.

Based on our testing on our existing datasets, we would recommend using the HRfunc tool for estimating channel-wise HRFs across a subject pool within fNIRS data and estimating neural signals. Even without an event-based design where HRF estimation is not possible, the HRfunc tool may be used to localize pre-existing estimates to a new fNIRS montage, given the quick and accurate spatial search capabilities of the tree data structure. This could be useful for resting state studies or block-based task designs where HRF estimation is not possible. The HRfunc tool can also be used for quick lookup of contextually relevant estimates using a hash table. We recommend the tool for exploring previous HRF estimates that may fit the contexts of the investigator’s own study and experiment. An investigator can also analyze ROIs to gain a higher accuracy HRF estimate through the HRfunc tool. This analysis can be conducted through the implemented radius search discussed in Sec. 2.2.4, or through a custom user-designed search algorithm centered around the k-d tree structure, such as a box search.

An important source of bias inherent to this tool’s methodology that one must consider is the use of relative hemoglobin compared with absolute concentration measures. Within HRfunc, deconvolution partially mitigates amplitude scaling bias by being scale invariant when estimating HRFs and neural activity. Although this approach reduces bias within the amplitude of our HRFs, risk of systematic bias still exists because some temporal individual variability in hemodynamic responses may not be captured in a group-level estimate. In practice, this means that although neural activity estimates may be more stable, they will still encounter issues with misalignment in time or shape for subjects whose HRF deviates from the group average.

A strategic approach to mitigating this bias is to estimate HRFs and neural activity in distinct subpopulations that represent a unique HRF distribution. Segmenting the subject pool into subpopulations may reduce biases introduced through assuming consistency between subjects’ hemodynamic responses. By estimating HRFs for unique populations in a subject pool, we may be obtaining a closer approximation of an HRF that better aligns with a population’s hemodynamics. As a result, HRF estimates will have fewer subjects to be estimated from as we further separate unique populations in a subject pool. This introduces a delicate tradeoff between specificity, accuracy, and power of our HRF estimates.

Consequently, HRfunc may not work well when data quality is low, sample size is minimal,^40^ or when one cannot estimate HRFs, such as in nonevent-based task designs.^58^ If the fNIRS data are of poor quality, the latent HRF and neural signal may not be present in sufficient quantities to estimate.^40^ In addition, if the sample size is too small, there may not be sufficient occurrences of an HRF present in the data to successfully recover the HRF from the signal.^42^^,^^58^ In both cases, an inaccurate HRF estimate may be calculated. The HRtree can be used to find previously estimated HRFs from other studies that are spatially and contextually similar from a higher quality dataset. However, the likelihood of finding similar HRFs will be affected by the current size of the HRtree storing estimates, contributed by users, and overall adoption of HRfunc by the fNIRS community.

Our goal is that in the future, the HRfunc tool will enable mass collaboration and communication of modeled HRF variability across studies. We see this tool as having potential to aid the field in reaching consistency and standardization in methodology^59^^,^^60^ through transparent HRF sharing, as described in Sec. 2.1.3. Through this collective resource neural activity can be deconvolved in fNIRS data where HRF estimation wasn’t possible in the past, or alternatively enables higher accuracy HRF and neural activity recovery through collaborative modeling of HRFs. Leveraging these collective resources as a database, we expect to analyze HRF variability on a larger scale across subjects and context for specific ROIs. Every additional HRF estimate added to the montage will allow us to create higher accuracy ROI HRF estimates across multiple montages and will enable us to model HRFs across contexts. HRfunc can also be used as a starting point for higher accuracy and more computationally expensive methods of HRF and neural signal estimation. For example, a Toeplitz deconvolved HRF from HRfunc can be used as a semi-known prior to Bayesian deconvolution^61^ to save computing time and resources. We expect to incorporate Bayesian deconvolution, alongside other methods, directly into the HRfunc tool in a future update.

## 5 Conclusion and Future Directions

Our results demonstrate that utilizing a local probabilistic HRF, through the newly developed HRfunc tool, enables more flexible and accurate recovery of neural signals, addressing major challenges existing in fNIRS preprocessing, such as temporal distortion and HRF variability. Although the current study focused on task-evoked and block-designed experiment data, leveraging the tree and hash table data structure in future use of the tool could be directed toward preprocessing signals from resting-state scans. Future efforts will explore meta-analysis of community-sourced HRFs and the effect experimental context has on HRF temporal profile and variability within fNIRS. Cross-compatibility with fMRI will be integrated into the HRfunc tool through coregistration^62^ to enable the wider neuroimaging community to better model and communicate hemodynamics across modalities. By enabling more accurate and shareable HRF estimates, this tool enhances the interpretability of fNIRS data for neuroscience and machine learning applications.

## Supplementary Material

10.1117/1.NPh.13.S1.S17801.s01

## Data Availability

The HRfunc library is published via the Python Packaging Index (PyPI), which can be downloaded via pip through any Python version 3.9.0 or greater. The code is hosted through GitHub at the repository github.com/dennys246/hrfunc; contributions to the repository are welcome. Guides for using the various HRfunc tool functionalities, alongside a form for submitting HRF estimates to the HRtree, are available at www.hrfunc.org. The P-CAT and CARE datasets used for estimating HRF’s and neural signals for evaluation of the HRfunc tool are available through the National Institutes of Mental Health Data Archive (NDA) upon request.
