# Supplementary material for: HRfunc: a tool for modeling hemodynamic response variability in fNIRS
Source: Neurophotonics. 2025 Nov 20;13(Suppl 1):S17801. doi: 10.1117/1.NPh.13.S1.S17801 (PMC12634216; doi:10.1117/1.NPh.13.S1.S17801)
Supplement: Supplementary file 1 [file NPh_013_S17801_SD001.pdf]

Paper	Journal	Author	Year	Purpose
The Circulation of Blood in the Human Brain		A. Mosso	1881	Showed through non-invasive means that brain activity is coupled to changes in blood flow.
On the regulation of the blood supply of the brain	Journal of Physiology	C. Roy & C. Sherrington	1890	Demonstrated experimentally that neuronal activity is coupled locally to blood flow and not fixed.
The Magnetic Properties and Structure of Hemoglobin, Oxyhemoglobin and Carbonmonoxyhemoglobin	Proceedings of the National Academy of Sciences	L. Pauling & C. Coryell	1936	Discovered deoxygenated hemoglobin is paramagnetic and local field distortions allowed us to detect quantity compared to oxygenated hemoglobin.
Brain magnetic resonance imaging with contrast dependent on blood oxygenation.	Proceedings of the National Academy of Sciences	Ogawa et al.	1990	Demonstrated that the magnetic susceptibility difference between oxygenated and deoxygenated hemoglobin could be exploited as a contrast mechanism in MRI.
The Relationship between Global and Local Changes in PET Scans	Journal of Cerebral Blood Flow & Metabolism	Friston et al.	1990	Formalized the use of convolution models to estimate neural activity from fMRI data, treating the HRF as a linear time-invariant system linking neuronal events to observed BOLD signals.
Dynamics of blood flow and oxygenation changes during brain activation: The balloon model	Magnetic Resonance in Medicine	Buxton et al.	1998	Introduced a biophysical 'Balloon model' describing how neural activity drives changes in cerebral blood flow, blood volume, and deoxyhemoglobin over time.
Deconvolution of Impulse Response in Event-Related BOLD fMRI	NeuroImage	G. Glover	1999	Applied deconvolution techniques to directly estimate the HRF from empirical BOLD data. Formalized the double-gamma HRF model.
Variation of BOLD hemodynamic responses across subjects and brain regions and their effects on statistical analyses	NeuroImage	Handwerker et al.	2004	Showed that HRFs can vary across brain regions and between people, which can ultimately cause problems in analysis.

Figure S1: A table summary of literature reviewing critical research that enabled the development of HRfunc.

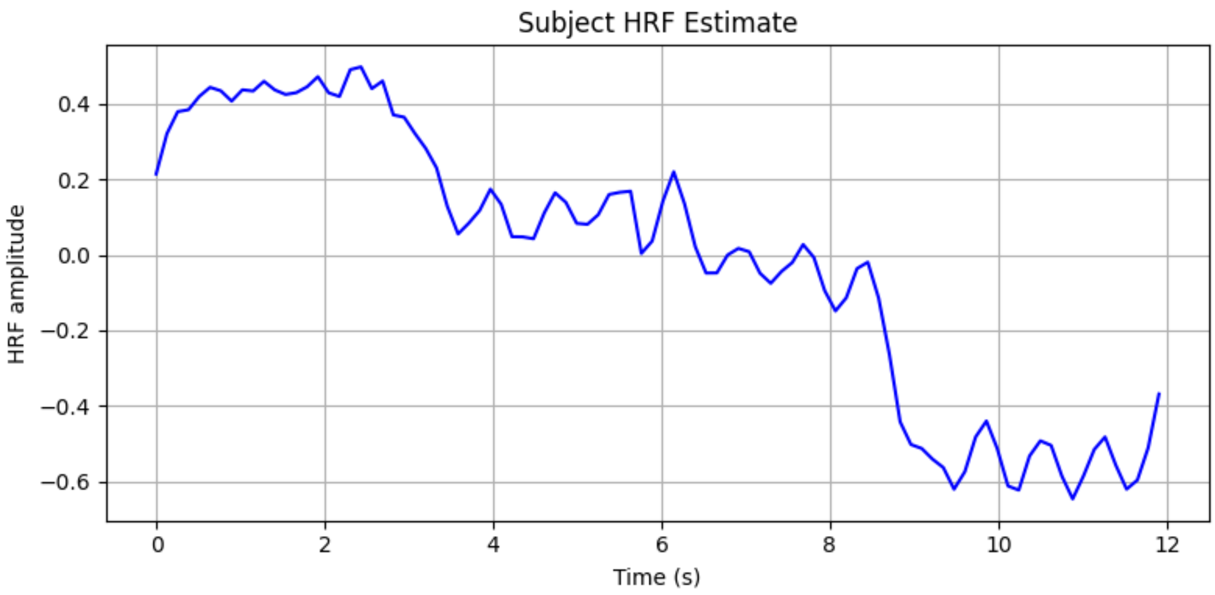

Figure S2: Single subject HRF estimated from the P-CAT Flanker task, deconvolved from a HbO fNIRS channel.

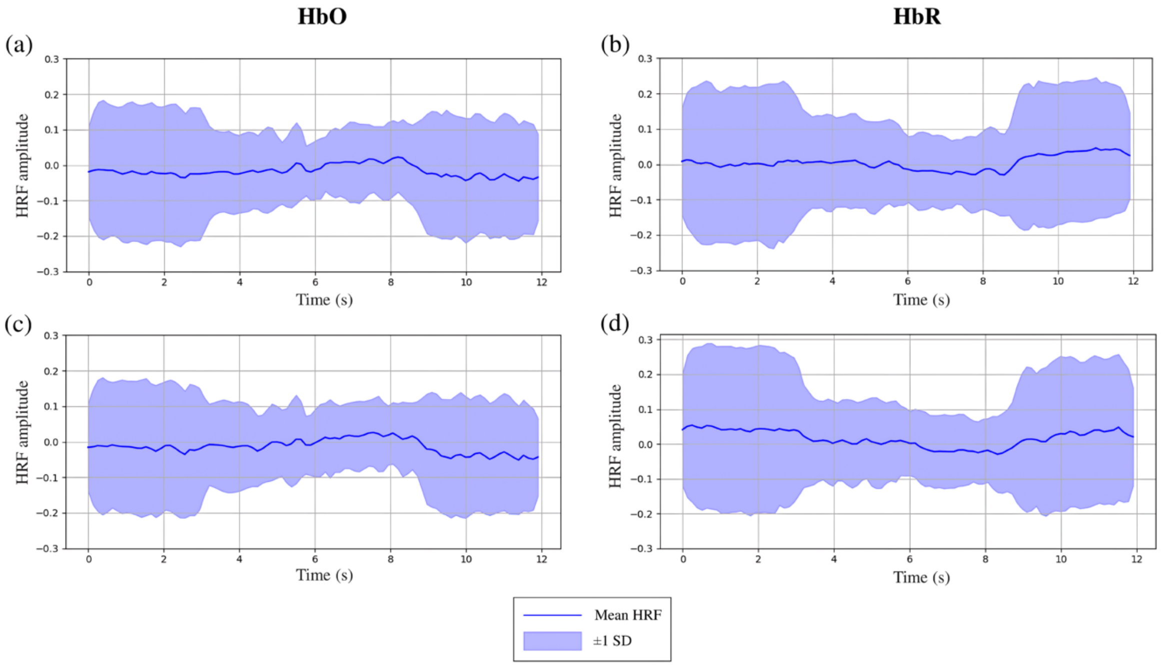

Figure S3: Examples of Toeplitz edge artifacts in HRF estimates from oxygenated (HbO) channels (a) and (c) alongside deoxygenated channel (HbR) estimates (b) and (d) with no edge expansion.
